# Posterior First and Second Cervical Vertebrae Fusion by Screw Fixation Technique using the Modern Pre-fabricated Template Method on Cadaver Samples

**DOI:** 10.5704/MOJ.2111.009

**Published:** 2021-11

**Authors:** M Athari, MR Golbakhsh, A Mirbolook, M Athari, A Ahmadi, K Komlakh, A Azarhomayoun, P Paydarniya

**Affiliations:** 1 Department of Orthopaedics, Shahid Beheshti University of Medical Sciences, Tehran, Iran; 2 Department of Orthopaedics, Tehran University of Medical Sciences, Tehran, Iran; 3 Department of Neurosurgery, Shahid Beheshti University of Medical Sciences, Tehran, Iran; 4 Department of Neurosurgery, Sina Trauma and Surgery Research Center, Tehran, Iran; 5 Department of Neurosurgery, Tehran University of Medical Sciences, Tehran, Iran

**Keywords:** atlas, axis, three-dimensional, fusion, pre-fabricated template

## Abstract

**Introduction:**

C1 lateral mass and C2 pedicular screws insertion are used for C1-C2 posterior fusion. Fluoroscopy Guided technique is routinely used for screw placement but it is associated with risk of injury to spinal cord and vertebral artery. 3D printing has developed rapidly in the fields of medicine. It is helpful in improving precise treatment and used for instrumentation in spine. We want to evaluate the accuracy of C1 lateral mass screws and C2 pedicle screws insertion by Pre-Fabricated Template made by three-dimensional (3D) printing.

**Materials and methods:**

Five cervical samples were obtained from cadavers. Based on fine-cut CT scan 3D-images reconstructed and the path of the screws designed by special software. A template produced by 3D-printer from 3D images. After printing the templates, they were fixed on the relevant vertebra in the operation room and based on the template path, C1 lateral mass screw and C2 pedicular screws were inserted. Placement of the screws was evaluated using CT scans post-operatively.

**Results:**

A total of 14 screws were inserted by above-mentioned method. After evaluation with CT scans none of the screws were entered in the spinal canal. Two screws had vertebral artery canal perforation with less than 50% breach. Violation was judged as noncritical and would probably not have resulted in injury to vertebral artery.

**Conclusions:**

The accuracy of C1 lateral mass screw and C2 pedicle screw insertion is acceptable with pre-fabricated template and can provide a useful aid for screw placement.

## Introduction

The first and second cervical vertebrae are among the most important and complex components of the vertebral column. The anatomy of these two vertebrae and their joints is one of the most complex and sensitive parts of the human body as they are connected by multiple ligaments and joints^1,2,3^. Similar to the other parts of the human body, this structure is prone to a number of diseases and injuries. A wide range of diseases, including inflammatory diseases, e.g., rheumatoid arthritis, congenital conditions, and trauma, can damage this structure leading to atlantoaxial instability^1-4^. The surgical treatment of atlantoaxial instability is performed by fixing the first and second vertebrae using a number of different methods. Among these methods, fixing with screws has been considered in recent years. The surgical treatment of this area is associated with high risk, even if it is performed by the most experienced surgeons, due to the complexity of the anatomy and the sensitivity of the elements in this area. Also, there is several anatomic variations in the general population.

The 3D printing has been employed in many surgical and medical fields. Such may include surgical planning, printing of models to aid with intra-operative guidance and anatomy definition, prosthesis development, medical education, patient-specific implant creation, and numerous other purposes^5^.

Due to the sensitivity of the atlantoaxial area and the complexity of the anatomy, it seems that pre-fabricated templates, which can guide the correct screw fixation path according to each person’s anatomy, can reduce the risk of surgery and its adverse consequences. The main objective of this study is to investigate the screw fixation technique for a C1 lateral mass screw and a pedicle screw in the second vertebra through pre-fabricated templates on cadaver samples.

## Materials and Methods

**Fig. 1:**
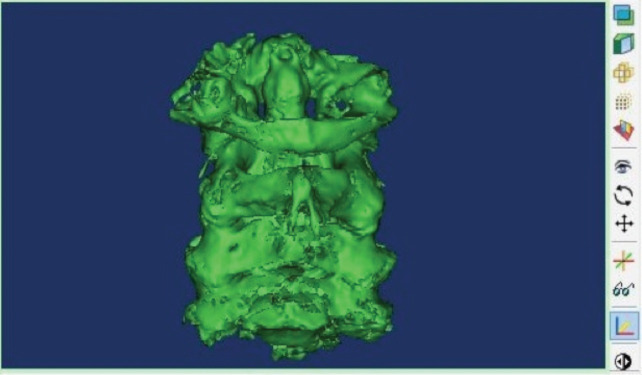
Medical Mimics Software Environment. This software is able to detect bone tissues according to the assumptions of the software, and it can perform a three-dimensional reconstruction.

At first, five cervical samples were obtained from the Anatomy Department of the University of Tehran. After numbering the samples, CT scan was performed with a delicate cut of 2mm (130 kv, 2mAs) at Sina Hospital Imaging Centre. The CT scan data were transferred to the three-dimensioning mimics software [Materialize Mimic software version 19] in the form of DICOM files for preparing Three-Dimensioning CT Scans. In this software, the CT scans taken from the samples are reconstructed in three-dimensional form. The software environment is shown in (Fig. 1).

**Fig. 2:**
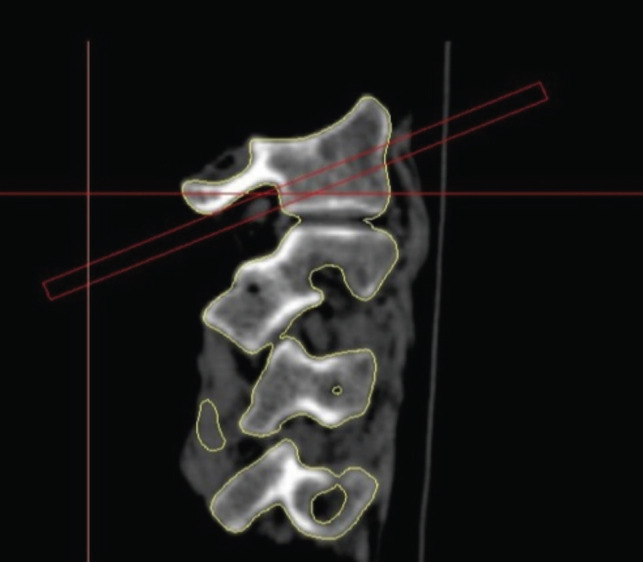
Sagittal CT from first vertebrae used for surgical planning.

Then, in the same environment, the correct direction of the screws was determined using cylinders in the software environment. The path for each cylinder was followed in all cuts of axial, coronal and sagittal planes of the CT scan, where each path of the cylinder was in its correct path. None of the cylinders had entered the canal or towards the holes of the vertebral artery (Fig. 2).

**Fig. 3:**
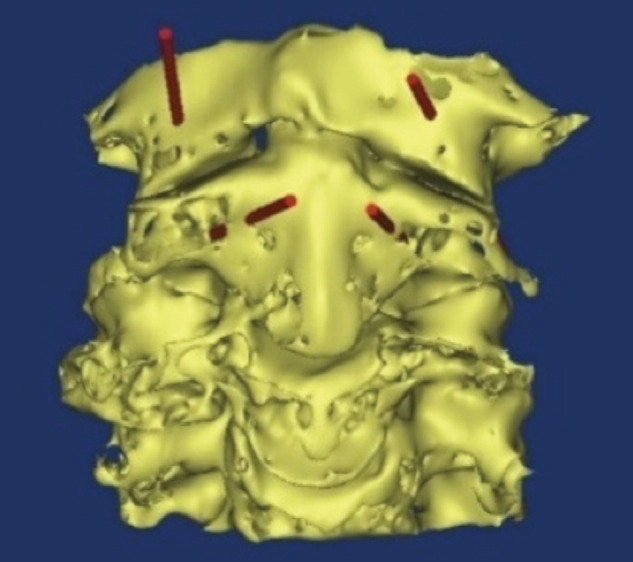
Three-dimensional coronal view. Red cylinders represent the path of the screws.

**Fig. 4:**
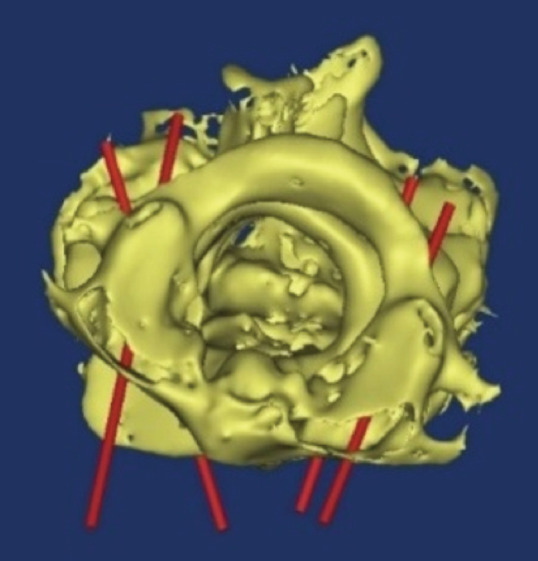
Three-dimensional axial view. Red cylinders represent the path of the screws.

**Fig. 5:**
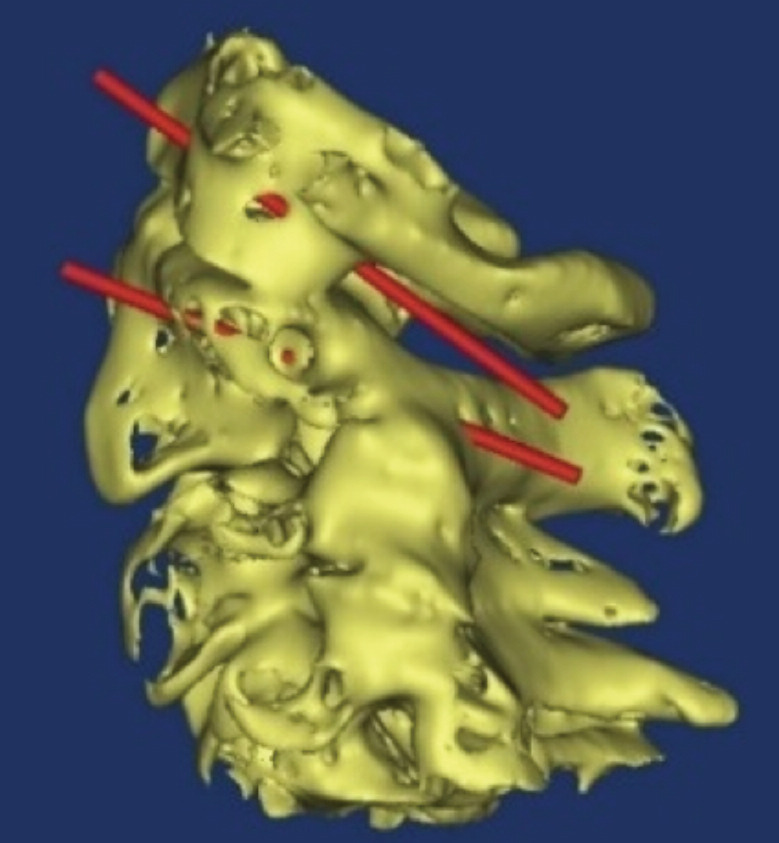
Three-dimensional axial view. Red cylinders represent the path of the screws.

With regards to the first and second cervical vertebra, the path of the screw was determined for each sample and examined in various cuts in terms of correctness of the path. Finally, the path of the screws in the three-dimensional view was examined (Fig. 3,4,5).

**Fig. 6:**
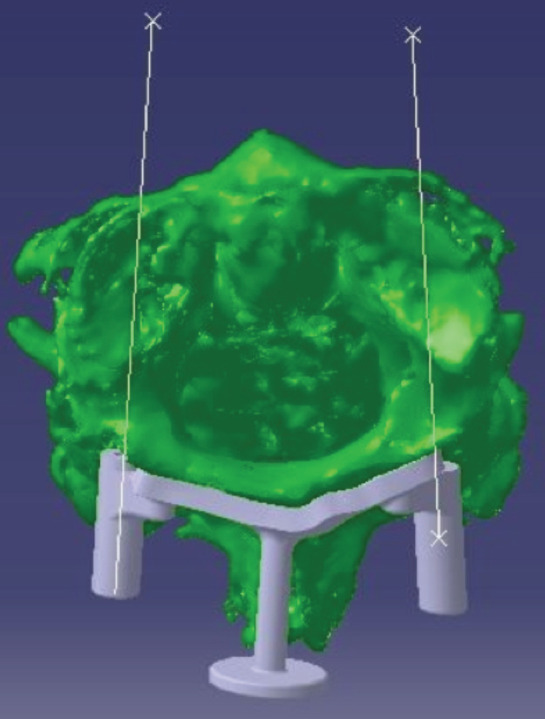
Templates designed based on the path of the screws. The lines represent the pre-specified path.

After designing the path of the screws, the information was stored as an STL file and transferred to the Geomatics Studio program. Then, based on the outer cortex and the posterior cortex of the first and second vertebrae, special templates were designed. These templates are perfectly fixed on the posterior section of each vertebra so that the path of the screws passes through the pre-fabricated templates (Fig. 6). Once the design stage was complete, the samples were prepared with the help of Jarah Yar Sharif Company (Centre for Science and Technology in Medicine, Imam Khomeini Hospital, Tehran, Iran) by printing them in three-dimensional form.

**Fig. 7:**
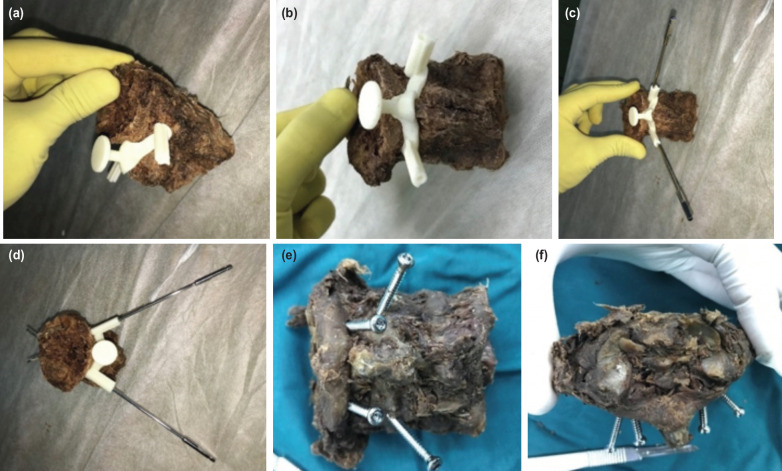
Stages of screw fixation and a screw- fixed sample. (a) The template fixed over the C2 vertebrae. (b) The template shown on the sample from different angle. (c) The path drilled in the path. (d) Same sample from different angle. (e) The screws inserted after drilling. (f) Same sample shown with the fixed screws.

After printing the templates, they were fixed on the relevant vertebra in the operation room at Sina Hospital. Then, based on the template path, a hole was created using a 2.7 drill. Next, a 3.5mm screw (Osweh company, Tehran, Iran) was fixed in the drilled position (Fig. 7).

After fixing the screws, the sites of the screws were checked by CT scan. In the performed CT scans, samples were examined in terms of extending into the spinal canal and the vertebral artery canal by Kaneyama *et al* classification^6^. The grades are as follows: Grade 1: None of the canals were damaged. Grade 2: Less than 50% of the screw is inside the canal. Grade 3: More than 50% of the screw is inside the canal.

## Results

**Fig. 8:**
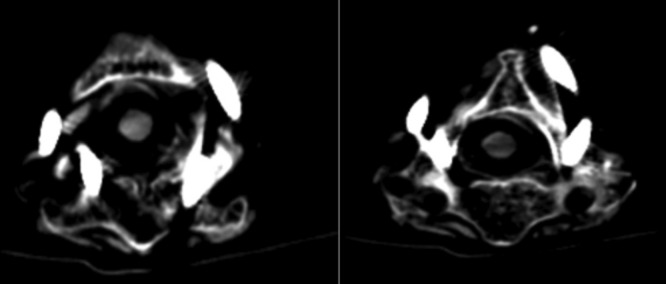
Post-operative CT scan for checking the screws’ site.

Four cervical samples were obtained from the anatomy department. Then, four screws were fixed in C1 and C2 of each sample according to the method described. Due to damage to the posterior arch of the cervical vertebra in one of the samples, it was impossible to place the screw in the first vertebra of this sample. Finally, 14 screws were fixed using the abovementioned method. Based on the post-operative CT scan (Fig. 8). the screws were ranked in three grades based on the extent of damage to the spinal canal and the vertebral arterial canal using Kaneyama *et al* classification for screw^6^.

In this study, none of the screws entered the spinal canal. Two screws were entered in vertebral artery canal that were grade 2, while none of the screws were in grade 3.

## Discussion

The fixation of a screw in the lateral masses of the first vertebra and the fixation of a screw inside the second vertebra’s pedicles require high skill and knowledge about the anatomy of this area. Surgery around theses area is difficult and dangerous due to the relatively small vertebral pedicle and the key anatomical structures around them. Therefore, comprehensive evaluation and precise navigation are very important for the surgery. Even with high levels of experience, there is a risk of damage to the spinal cord and the vertebral artery. The normal navigation methods face the problem of intra-operative drift; thus, the screw may easily break through the bone cortex when the pedicle is small^7-11^. Patient-specific drill guide templates have been developed as an inexpensive, accurate method to guide screw insertion in the spine based on advances in 3D computing and printing technologies. Ryken *et al* fixed screws on four cadaver samples for the first time using pre-fabricated templates^12^.

Moreover, D’urso *et al* showed that the specific template for each vertebra had high accuracy in screw fixation^13^. In addition, Van Cleynenbreugel *et al* used two types of templates for screw fixation using the Magerl method^14^. Hu *et al* used a pre-fabricated template for screw fixation using the Magerl method^15^. Sugawara *et al* used template for posterior C1-C2 fusion. they inserted a total of 48 screws with accuracy of 100%^16^. Guo *et al* compared C1 and C2 screw fixation in patients guided by the template and the patients which screws were fixed under guidance of fluoroscopy. In the template guided group, the acceptability rate was 94.6% compared to 70.27% in control group. The acceptability rate in test group was higher than the rate in control group. The operation time and fluoroscopic frequency for each screw were decreased, compared with the control group^17^.

In another study comparing a total of 68 screws placed at the pedicle in the 19 patients who underwent atlantoaxial pedicle screw assisted by a 3D-printed guide plate, while a total of 76 screws were placed at the pedicle in 24 patients using the traditional method. The accuracy of screw placement in the 3D-printed plate group (94.1%) was far higher than that in the traditional fixation group (76.3%). atlantoaxial pedicle screw fixation assisted by a 3D-printed plate significantly improved the accuracy rate of screw placement^18^. The navigation template used in the current study was built by several reverse engineering software packages; thus, it required manual adjustments. These factors affect the accuracy of the method, and different studies report different navigation accuracy rates (i.e., 84-97.6%)^19-22^.

Pre-fabricated samples based on the patient’s CT scans try to reduce the error during the surgery and minimise adverse complications of these operations. The screw fixation technique based on pre-fabricated templates is a modern technique, which reduces the risk of such operations. The results of the current study are comparable to the findings of the previous studies. However, the screws damaged the cortex of the vertebral canal in two cases, which may have been due to the very small diameter of the samples’ pedicle. Therefore, the screw fixation method is not recommended if the diameter of the pedicle is small.

To our knowledge, this is the first study in Iran for assessment of feasibility and accuracy of a 3D-printed template for spinal instrumentation. The first limitation of this study is the low number of cadaver samples. Unfortunately, access to cadaver samples has many problems in our country and samples often do not have good quality. The soft tissue of these cadaver samples does not have good consolidation. In addition, samples undergo changes in bone density due to long-term storage, which are evident in the CT scans and differ from living samples. Another limitation of this study is that common screws were used, due to the high cost of cervical screws, and these common screws cause artifacts in the post-operative CT scans.

As such, we expect to summarise the advantages and disadvantages of the 3D printing technology in a follow-up work in vivo and promote the relevant clinical applications.

## Conclusion

Cervical pedicle screw placement using a rapid prototyping drill template based on a 3D model is technically feasible, and it can enable accurate pedicle screw placement without the need for extra radiation. This study indicates the ability to perform such techniques in Iran; however, further studies are needed in this area.
